# 1-(4-Bromo­phen­yl)-2-(phenyl­sulfon­yl)ethanone

**DOI:** 10.1107/S1600536811036671

**Published:** 2011-09-14

**Authors:** Hatem A. Abdel-Aziz, Seik Weng Ng, Edward R. T. Tiekink

**Affiliations:** a Department of Pharmaceutical Chemistry, College of Pharmacy, King Saud University, Riyadh 11451, Saudi Arabia; b Department of Chemistry, University of Malaya, 50603 Kuala Lumpur, Malaysia; c Chemistry Department, Faculty of, Science, King Abdulaziz University, PO Box 80203 Jeddah, Saudi Arabia

## Abstract

The overall conformation of the title mol­ecule, C_14_H_11_BrO_3_S, is L-shaped, as seen in the value of the dihedral angle formed between the terminal benzene rings of 75.44 (13)°. The presence of C—H⋯O inter­actions leads to the formation of linear supra­molecular chains along the *a*-axis direction in the crystal structure. These are connected into supra­molecular arrays in the *ab* plane *via* C—H⋯π contacts.

## Related literature

For the biological activity of sulphones, see: Garuti *et al.* (2002 ▶); Abdel-Aziz & Mekawey (2009 ▶); Abdel-Aziz *et al.* (2010 ▶). For the synthesis, see: Takahashi *et al.* (1986 ▶).
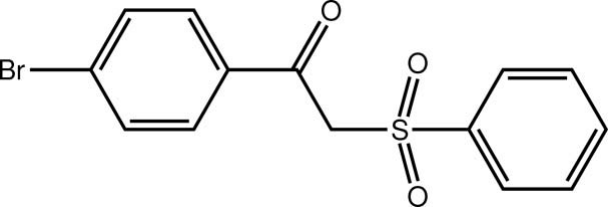


## Experimental

### 

#### Crystal data

C_14_H_11_BrO_3_S
*M* *_r_* = 339.20
Triclinic, 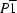

*a* = 5.6467 (4) Å
*b* = 10.3597 (6) Å
*c* = 11.1934 (6) Å
α = 86.430 (5)°
β = 89.177 (5)°
γ = 83.763 (5)°
*V* = 649.64 (7) Å^3^
*Z* = 2
Cu *K*α radiation
μ = 5.83 mm^−1^
*T* = 100 K
0.25 × 0.20 × 0.02 mm

#### Data collection

Agilent SuperNova Dual diffractometer with Atlas detector
Absorption correction: multi-scan (*CrysAlis PRO*; Agilent, 2010 ▶) *T*_min_ = 0.291, *T*_max_ = 1.000
4181 measured reflections
2533 independent reflections
2396 reflections with *I* > 2σ(*I*)
*R*_int_ = 0.034

#### Refinement

*R*[*F* ^2^ > 2σ(*F* ^2^)] = 0.039
*wR*(*F* ^2^) = 0.108
*S* = 1.04
2533 reflections
172 parameters
H-atom parameters constrained
Δρ_max_ = 0.69 e Å^−3^
Δρ_min_ = −0.80 e Å^−3^

### 

Data collection: *CrysAlis PRO* (Agilent, 2010 ▶); cell refinement: *CrysAlis PRO*; data reduction: *CrysAlis PRO*; program(s) used to solve structure: *SHELXS97* (Sheldrick, 2008 ▶); program(s) used to refine structure: *SHELXL97* (Sheldrick, 2008 ▶); molecular graphics: *ORTEP-3* (Farrugia, 1997 ▶) and *DIAMOND* (Brandenburg, 2006 ▶); software used to prepare material for publication: *publCIF* (Westrip, 2010 ▶).

## Supplementary Material

Crystal structure: contains datablock(s) global, I. DOI: 10.1107/S1600536811036671/pv2446sup1.cif

Structure factors: contains datablock(s) I. DOI: 10.1107/S1600536811036671/pv2446Isup2.hkl

Supplementary material file. DOI: 10.1107/S1600536811036671/pv2446Isup3.cml

Additional supplementary materials:  crystallographic information; 3D view; checkCIF report

## Figures and Tables

**Table 1:** Hydrogen-bond geometry (Å, °) *Cg*1 is the centroid of the C9–C14 ring.

*D*—H⋯*A*	*D*—H	H⋯*A*	*D*⋯*A*	*D*—H⋯*A*
C7—H7a⋯O1^i^	0.99	2.29	3.250 (3)	162
C7—H7b⋯O1^ii^	0.99	2.47	3.307 (3)	142
C4—H4⋯*Cg*1^iii^	0.95	2.87	3.708 (3)	147

Symmetry codes: (i) 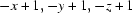; (ii) 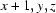; (iii) 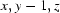.

**Fig. 1.**
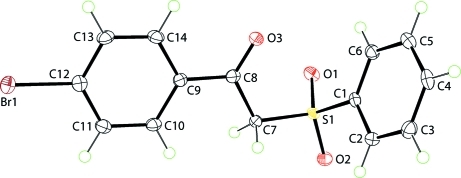
The molecular structure of the title compound showing the atom-labelling scheme and displacement ellipsoids at the 50% probability level.

**Fig. 2.**
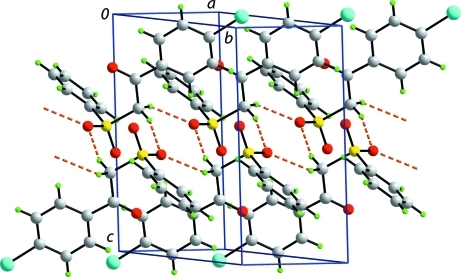
Supramolecular chain of the title molecules mediated by C—H···O interactions, shown as orange lines.

**Fig. 3.**
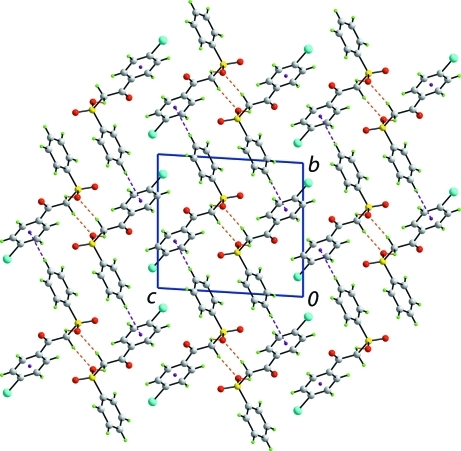
A view in projection down the a axis of the unit-cell contents of the title compound. The C—H···O and C—H···π interactions are shown as orange and purple dashed lines, respectively.

## supplementary crystallographic information

### Comment

The analysis of the title compound, was prompted by the biological activity displayed by sulphones (Garuti, *et al.*, 2002; Abdel-Aziz & Mekawey, 2009; Abdel-Aziz *et al.*, 2010).

In the title molecule (Fig. 1), both sulfonyl-O atoms lie to one side of the S-bound benzene ring, and the methylene group to the other. The dihedral angle formed between the benzene rings of 75.44 (13) ° is consistent with the molecule having an overall *L*-shape.

Supramolecular chains aligned along the *a* axis are the most predominant feature of the crystal packing (Table 1 and Fig. 2). Molecules are connected into a linear chain by an alternating sequence of centrosymmetric eight-membered {···HCH···O}_2_ and {···HCSO}_2_ synthons. Chains are linked into layers in the *ab* plane by C—H···π involving the S-bound and Br-benzene rings as donors and acceptors, respectively.

### Experimental

The title compound was prepared according to the reported method (Takahashi *et al.*, 1986). The crystals were isolated from its EtOH/DMF (*v*/*v* = 5/1) solution by slow evaporation at room temperature.

### Refinement

Carbon-bound H-atoms were placed in calculated positions [C—H 0.95 to 0.99 Å, *U*_iso_(H) 1.2*U*_eq_(C)] and were included in the refinement in the riding model approximation.

### Crystal data

**Table:**

C_14_H_11_BrO_3_S	*Z* = 2
*M**_r_* = 339.20	*F*(000) = 340
Triclinic, *P*1	*D*_x_ = 1.734 Mg m^−^^3^
Hall symbol: -P 1	Cu *K*α radiation, λ = 1.5418 Å
*a* = 5.6467 (4) Å	Cell parameters from 2772 reflections
*b* = 10.3597 (6) Å	θ = 4.0–74.2°
*c* = 11.1934 (6) Å	µ = 5.83 mm^−^^1^
α = 86.430 (5)°	*T* = 100 K
β = 89.177 (5)°	Plate, light-brown
γ = 83.763 (5)°	0.25 × 0.20 × 0.02 mm
*V* = 649.64 (7) Å^3^	

### Data collection

**Table:**

Agilent SuperNova Dual diffractometer with Atlas detector	2533 independent reflections
Radiation source: SuperNova (Cu) X-ray Source	2396 reflections with *I* > 2σ(*I*)
mirror	*R*_int_ = 0.034
Detector resolution: 10.4041 pixels mm^-1^	θ_max_ = 74.4°, θ_min_ = 4.0°
ω scan	*h* = −7→7
Absorption correction: multi-scan (*CrysAlis PRO*; Agilent, 2010)	*k* = −12→6
*T*_min_ = 0.291, *T*_max_ = 1.000	*l* = −13→13
4181 measured reflections	

### Refinement

**Table:**

Refinement on *F*^2^	Primary atom site location: structure-invariant direct methods
Least-squares matrix: full	Secondary atom site location: difference Fourier map
*R*[*F*^2^ > 2σ(*F*^2^)] = 0.039	Hydrogen site location: inferred from neighbouring sites
*wR*(*F*^2^) = 0.108	H-atom parameters constrained
*S* = 1.04	*w* = 1/[σ^2^(*F*_o_^2^) + (0.0821*P*)^2^ + 0.0577*P*] where *P* = (*F*_o_^2^ + 2*F*_c_^2^)/3
2533 reflections	(Δ/σ)_max_ < 0.001
172 parameters	Δρ_max_ = 0.69 e Å^−^^3^
0 restraints	Δρ_min_ = −0.80 e Å^−^^3^

### Special details

**Table:**

Geometry. All e.s.d.'s (except the e.s.d. in the dihedral angle between two l.s. planes) are estimated using the full covariance matrix. The cell e.s.d.'s are taken into account individually in the estimation of e.s.d.'s in distances, angles and torsion angles; correlations between e.s.d.'s in cell parameters are only used when they are defined by crystal symmetry. An approximate (isotropic) treatment of cell e.s.d.'s is used for estimating e.s.d.'s involving l.s. planes.
Refinement. Refinement of *F*^2^ against ALL reflections. The weighted *R*-factor *wR* and goodness of fit *S* are based on *F*^2^, conventional *R*-factors *R* are based on *F*, with *F* set to zero for negative *F*^2^. The threshold expression of *F*^2^ > σ(*F*^2^) is used only for calculating *R*-factors(gt) *etc*. and is not relevant to the choice of reflections for refinement. *R*-factors based on *F*^2^ are statistically about twice as large as those based on *F*, and *R*- factors based on ALL data will be even larger.

### Fractional atomic coordinates and isotropic or equivalent isotropic displacement parameters (Å^2^)

**Table:**

	*x*	*y*	*z*	*U*_iso_*/*U*_eq_	
Br1	1.22639 (4)	0.85297 (2)	−0.03616 (2)	0.01909 (14)	
S1	0.56382 (10)	0.30122 (6)	0.45101 (5)	0.01267 (17)	
O1	0.3196 (3)	0.36055 (18)	0.44650 (17)	0.0179 (4)	
O2	0.6741 (3)	0.27303 (18)	0.56677 (16)	0.0187 (4)	
O3	0.5107 (3)	0.40819 (18)	0.19816 (16)	0.0193 (4)	
C1	0.5826 (4)	0.1561 (2)	0.3754 (2)	0.0137 (5)	
C2	0.7865 (5)	0.0678 (3)	0.3911 (2)	0.0168 (5)	
H2	0.9129	0.0869	0.4399	0.020*	
C3	0.8018 (5)	−0.0480 (3)	0.3345 (2)	0.0198 (5)	
H3	0.9389	−0.1094	0.3449	0.024*	
C4	0.6160 (5)	−0.0744 (3)	0.2624 (3)	0.0195 (5)	
H4	0.6276	−0.1534	0.2228	0.023*	
C5	0.4139 (5)	0.0141 (3)	0.2480 (2)	0.0178 (5)	
H5	0.2877	−0.0051	0.1991	0.021*	
C6	0.3948 (4)	0.1304 (2)	0.3045 (2)	0.0164 (5)	
H6	0.2567	0.1911	0.2950	0.020*	
C7	0.7509 (5)	0.4054 (2)	0.3716 (2)	0.0137 (5)	
H7A	0.7601	0.4828	0.4182	0.016*	
H7B	0.9134	0.3591	0.3678	0.016*	
C8	0.6743 (4)	0.4514 (2)	0.2452 (2)	0.0147 (5)	
C9	0.8097 (4)	0.5513 (2)	0.1824 (2)	0.0143 (5)	
C10	1.0105 (5)	0.5959 (3)	0.2326 (2)	0.0158 (5)	
H10	1.0623	0.5639	0.3103	0.019*	
C11	1.1329 (5)	0.6872 (3)	0.1681 (2)	0.0173 (5)	
H11	1.2680	0.7183	0.2016	0.021*	
C12	1.0565 (5)	0.7321 (2)	0.0551 (2)	0.0162 (5)	
C13	0.8565 (5)	0.6902 (3)	0.0040 (2)	0.0184 (5)	
H13	0.8051	0.7228	−0.0736	0.022*	
C14	0.7355 (5)	0.6008 (3)	0.0685 (2)	0.0181 (5)	
H14	0.5984	0.5720	0.0349	0.022*	

### Atomic displacement parameters (Å^2^)

**Table:**

	*U*^11^	*U*^22^	*U*^33^	*U*^12^	*U*^13^	*U*^23^
Br1	0.0211 (2)	0.0184 (2)	0.01762 (19)	−0.00312 (12)	0.00239 (12)	0.00122 (12)
S1	0.0132 (3)	0.0128 (3)	0.0125 (3)	−0.0021 (2)	−0.0015 (2)	−0.0028 (2)
O1	0.0136 (9)	0.0173 (9)	0.0232 (9)	0.0003 (7)	0.0005 (7)	−0.0078 (7)
O2	0.0231 (10)	0.0203 (10)	0.0133 (9)	−0.0045 (7)	−0.0034 (7)	−0.0013 (7)
O3	0.0230 (9)	0.0170 (9)	0.0191 (9)	−0.0066 (7)	−0.0078 (7)	0.0003 (7)
C1	0.0163 (11)	0.0117 (11)	0.0139 (11)	−0.0044 (9)	0.0012 (9)	−0.0017 (8)
C2	0.0166 (12)	0.0173 (12)	0.0168 (12)	−0.0036 (10)	−0.0043 (9)	0.0008 (9)
C3	0.0198 (12)	0.0163 (13)	0.0226 (13)	0.0002 (10)	0.0012 (10)	0.0002 (10)
C4	0.0250 (14)	0.0144 (12)	0.0206 (12)	−0.0081 (10)	0.0056 (10)	−0.0046 (9)
C5	0.0178 (12)	0.0201 (13)	0.0171 (12)	−0.0075 (10)	0.0019 (9)	−0.0039 (9)
C6	0.0157 (12)	0.0164 (12)	0.0173 (12)	−0.0028 (9)	0.0001 (9)	−0.0009 (9)
C7	0.0151 (11)	0.0125 (11)	0.0138 (12)	−0.0026 (9)	−0.0024 (9)	−0.0011 (9)
C8	0.0169 (11)	0.0104 (11)	0.0164 (12)	0.0020 (9)	−0.0031 (9)	−0.0040 (9)
C9	0.0177 (12)	0.0108 (11)	0.0145 (12)	−0.0004 (9)	−0.0029 (9)	−0.0033 (9)
C10	0.0171 (12)	0.0169 (12)	0.0133 (11)	−0.0007 (9)	−0.0031 (9)	−0.0018 (9)
C11	0.0154 (12)	0.0182 (12)	0.0187 (13)	−0.0022 (9)	−0.0027 (9)	−0.0026 (10)
C12	0.0179 (12)	0.0145 (12)	0.0165 (12)	−0.0010 (9)	0.0019 (9)	−0.0039 (9)
C13	0.0228 (13)	0.0199 (13)	0.0125 (11)	−0.0011 (10)	−0.0052 (9)	−0.0012 (9)
C14	0.0183 (12)	0.0193 (12)	0.0175 (12)	−0.0035 (10)	−0.0058 (10)	−0.0031 (10)

### Geometric parameters (Å, °)

**Table:**

Br1—C12	1.899 (3)	C6—H6	0.9500
S1—O2	1.4452 (19)	C7—C8	1.518 (3)
S1—O1	1.4478 (18)	C7—H7A	0.9900
S1—C1	1.763 (2)	C7—H7B	0.9900
S1—C7	1.781 (3)	C8—C9	1.489 (4)
O3—C8	1.210 (3)	C9—C14	1.397 (3)
C1—C6	1.392 (3)	C9—C10	1.407 (4)
C1—C2	1.396 (3)	C10—C11	1.391 (4)
C2—C3	1.386 (4)	C10—H10	0.9500
C2—H2	0.9500	C11—C12	1.379 (4)
C3—C4	1.393 (4)	C11—H11	0.9500
C3—H3	0.9500	C12—C13	1.395 (4)
C4—C5	1.389 (4)	C13—C14	1.372 (4)
C4—H4	0.9500	C13—H13	0.9500
C5—C6	1.388 (4)	C14—H14	0.9500
C5—H5	0.9500		
			
O2—S1—O1	118.37 (11)	S1—C7—H7A	108.3
O2—S1—C1	108.48 (11)	C8—C7—H7B	108.3
O1—S1—C1	108.42 (11)	S1—C7—H7B	108.3
O2—S1—C7	104.73 (11)	H7A—C7—H7B	107.4
O1—S1—C7	109.49 (12)	O3—C8—C9	121.8 (2)
C1—S1—C7	106.77 (11)	O3—C8—C7	121.2 (2)
C6—C1—C2	121.6 (2)	C9—C8—C7	117.0 (2)
C6—C1—S1	120.05 (19)	C14—C9—C10	119.0 (2)
C2—C1—S1	118.32 (18)	C14—C9—C8	118.4 (2)
C3—C2—C1	119.1 (2)	C10—C9—C8	122.6 (2)
C3—C2—H2	120.5	C11—C10—C9	119.8 (2)
C1—C2—H2	120.5	C11—C10—H10	120.1
C2—C3—C4	119.9 (2)	C9—C10—H10	120.1
C2—C3—H3	120.1	C12—C11—C10	119.4 (2)
C4—C3—H3	120.1	C12—C11—H11	120.3
C5—C4—C3	120.4 (2)	C10—C11—H11	120.3
C5—C4—H4	119.8	C11—C12—C13	121.8 (2)
C3—C4—H4	119.8	C11—C12—Br1	120.0 (2)
C4—C5—C6	120.5 (2)	C13—C12—Br1	118.2 (2)
C4—C5—H5	119.7	C14—C13—C12	118.5 (2)
C6—C5—H5	119.7	C14—C13—H13	120.7
C5—C6—C1	118.5 (2)	C12—C13—H13	120.7
C5—C6—H6	120.8	C13—C14—C9	121.4 (2)
C1—C6—H6	120.8	C13—C14—H14	119.3
C8—C7—S1	115.92 (18)	C9—C14—H14	119.3
C8—C7—H7A	108.3		
			
O2—S1—C1—C6	142.5 (2)	S1—C7—C8—O3	−7.5 (3)
O1—S1—C1—C6	12.8 (2)	S1—C7—C8—C9	172.60 (17)
C7—S1—C1—C6	−105.1 (2)	O3—C8—C9—C14	3.6 (3)
O2—S1—C1—C2	−36.0 (2)	C7—C8—C9—C14	−176.6 (2)
O1—S1—C1—C2	−165.7 (2)	O3—C8—C9—C10	−175.5 (3)
C7—S1—C1—C2	76.4 (2)	C7—C8—C9—C10	4.4 (3)
C6—C1—C2—C3	0.1 (4)	C14—C9—C10—C11	−0.6 (4)
S1—C1—C2—C3	178.6 (2)	C8—C9—C10—C11	178.4 (2)
C1—C2—C3—C4	0.5 (4)	C9—C10—C11—C12	−0.5 (4)
C2—C3—C4—C5	−0.8 (4)	C10—C11—C12—C13	1.1 (4)
C3—C4—C5—C6	0.5 (4)	C10—C11—C12—Br1	−178.25 (18)
C4—C5—C6—C1	0.1 (4)	C11—C12—C13—C14	−0.7 (4)
C2—C1—C6—C5	−0.4 (4)	Br1—C12—C13—C14	178.7 (2)
S1—C1—C6—C5	−178.86 (19)	C12—C13—C14—C9	−0.5 (4)
O2—S1—C7—C8	−179.65 (18)	C10—C9—C14—C13	1.1 (4)
O1—S1—C7—C8	−51.8 (2)	C8—C9—C14—C13	−178.0 (2)
C1—S1—C7—C8	65.4 (2)		

### Hydrogen-bond geometry (Å, °)

**Table:**

Cg1 is the centroid of the C9–C14 ring.

**Table:**

*D*—H···*A*	*D*—H	H···*A*	*D*···*A*	*D*—H···*A*
C7—H7a···O1^i^	0.99	2.29	3.250 (3)	162
C7—H7b···O1^ii^	0.99	2.47	3.307 (3)	142
C4—H4···Cg1^iii^	0.95	2.87	3.708 (3)	147

Symmetry codes: (i) −*x*+1, −*y*+1, −*z*+1; (ii) *x*+1, *y*, *z*; (iii) *x*, *y*−1, *z*.
